# A scoping review of microbiological safety, bioactive components, nutrient retention, and feasibility of flash-heating expressed breastmilk: exploring potential use during infectious disease outbreaks

**DOI:** 10.3389/fnut.2026.1926806

**Published:** 2026-10-15

**Authors:** Helen Natalia, Carolina Guzman, Bindi Borg, Jodine Chase, Anna Coutsoudis, Kiersten Israel-Ballard, Karleen Gribble, Peggy Koniz-Booher, Mija Ververs

**Affiliations:** 1 Department of Health, Behavior and Society, Johns Hopkins Bloomberg School of Public Health, Baltimore, MD, United States; 2 Department of International Health, Johns Hopkins Bloomberg School of Public Health, Baltimore, MD, United States; 3 Health Research Institute, Faculty of Health, University of Canberra, Canberra, ACT, Australia; 4 SafelyFed Canada, Mississauga, ON, Canada; 5 Department of Paediatrics and Child Health, College of Health Sciences, University of KwaZulu-Natal, Durban, South Africa; 6 Department of Global Health, University of Washington, Seattle, WA, United States; 7 School of Nursing and Midwifery, Faculty of Health, Western Sydney University, Sydney, NSW, Australia; 8 Consultant, Baltimore, MD, United States; 9 Center for Humanitarian Health, Johns Hopkins Bloomberg School of Public Health, Baltimore, MD, United States

**Keywords:** breastmilk, flash-heating, infectious diseases, outbreaks, pasteurization

## Abstract

**Introduction:**

Infectious disease outbreaks can disrupt breastfeeding due to concerns about pathogen transmission through close contact or breastmilk. In these contexts, infants are often fed commercial milk formula or other breastmilk substitutes despite their known risks, which are exacerbated in low-resource settings. Existing technologies to inactivate pathogens in human milk, such as holder and high-temperature short-time (HTST) pasteurization, require specialized equipment and may not be feasible in emergency settings. Flash-heating (FH) is a low-cost, household-level alternative that approximates HTST conditions and can inactivate pathogens while preserving key nutritional and immunological components. This review examines the evidence on FH as a potential method for pasteurizing expressed breastmilk during infectious disease outbreaks.

**Methods:**

This scoping review followed the Joanna Briggs Institute guidelines and the PRISMA-ScR checklist. Searches were performed in PubMed, Scopus, CINAHL, Embase, and Web of Science, supplemented by grey literature and forward citation searching. Studies on FH of expressed human milk were included if they evaluated on (1) microbiological safety, (2) preservation of nutritional and immunological components, and (3) feasibility in low-resource and outbreak settings. Two independent reviewers screened studies and extracted data using Covidence, and the evidence were descriptively synthesized.

**Results:**

A total of 24 studies published between 2005 and 2025 were included. FH is effective in inhibiting growth of a range of bacterial and viral pathogens. Effectiveness varies by pathogen type and heat sensitivity. FH preserves substantial proportions of key nutrients and immunological components in human milk, such as immunoglobulins, lactoferrin, vitamins, and oligosaccharides. Research and operational gaps remain regarding standardization, feasibility, and acceptability in real-world outbreak contexts.

**Conclusion:**

Flash-heating shows potential as an option for enabling infants to be fed maternal breastmilk when direct breastfeeding and use of untreated breastmilk is not safe. Strengthening the evidence base will be essential to inform clear, context-appropriate guidance and support safe infant feeding practices during future outbreaks.

## 1 Introduction

Over the past decades, multiple infectious disease outbreaks have led to recommendations for the temporary cessation of breastfeeding due to concerns regarding the potential transmission of pathogens through close physical contact (1). Infants in these situations have often been fed commercial milk formula (CMF) or other breastmilk substitutes (BMS), despite well-documented risks associated with artificial feeding, which are exacerbated in low-resource settings (2). These risks include increased rates of diarrheal disease, respiratory tract infections, and overall infant morbidity and mortality (3–5). Infection control guidance has also sometimes recommended against feeding expressed breastmilk to infants due to concerns about possible disease transmission via the milk itself. However, evidence is usually initially insufficient to determine whether a newly emerging pathogen is present or viable in human milk. The COVID-19 pandemic highlighted these uncertainties, as limited early evidence and contradictory evolving guidance contributed to recommendations against breastfeeding and feeding expressed breastmilk (6). This reflected a misapplication of the precautionary principle, and lacked proper weighing of the uncertain risks of pathogen transmission and evidently low risk of severe disease against the well-established harms of artificial feeding (7).

In the context of a novel infectious disease outbreak, heat treatment of expressed breastmilk provides potential for preventing disease transmission if breastmilk is deemed a significant risk to infants. Several technologies have been explored over the years to provide breastmilk rendered free of pathogens. These include Holder pasteurization, high-temperature short-time (HTST) pasteurization, high-pressure processing, ultraviolet radiation, and use of pulsed electric fields. Among these, thermal methods such as Holder pasteurization and HTST have been used in milk banking. Holder pasteurization involves ‘holding’ the milk at 62.5 °C for 30 min and is the most commonly used approach for pasteurizing donor human milk in human milk banks. HTST pasteurization, which utilizes a continuous flow system to achieve 72 °C for 15 s, is widely used in the bovine dairy industry and is increasingly being explored using adapted technologies in a number of human milk banks in high-resource settings. While effective at pathogen growth inhibition, these methods require specialized equipment and processing systems and may not be feasible in low-resource, household-level or emergency contexts (8).

Flash-heating (FH) was developed over two decades ago to provide HIV positive mothers in resource-limited settings an option for eliminating the risk of HIV transmission to their infants via their breastmilk (9). FH is a low-technology, inexpensive technique for human milk pasteurization that aims to mimic the HTST heating process but using a more simplified approach with common household equipment. In this method, 50–120 mL of milk is placed in an uncovered 450 mL glass food jar, which is then placed in approximately 450 mL of water inside a 1–2 L pan ensuring that the water level is two fingers above the level of milk. The water and milk are heated simultaneously over an electric hot plate or gas stove until the water reaches a rolling, or vigorous, boil (100 °C); after that, the glass jar of milk is immediately removed and rapidly cooled in a cool water bath (10, 11). FH has been shown to inhibit growth of some pathogens while preserving a substantial proportion of the nutritional and immunological components of human milk (9).

Although important knowledge gaps remain, FH represents a potentially useful, but underexamined, practical strategy in outbreak settings where there are concerns about pathogen transmission through breastmilk and access to adequately safe alternatives is limited. The effectiveness of thermal treatments such as FH are likely to vary depending on the type of pathogen, particularly between bacterial and viral agents with differing heat sensitivities. In addition, the extent to which these treatments preserve the nutritional and immunological quality of breastmilk may vary across conditions. Finally, although intended to approximate HTST, FH does not achieve the same temperature profile (8, 10–12).

This review aims to examine the existing evidence on FH as a strategy for managing expressed breastmilk during infectious disease outbreaks, with evidence from HTST studies included to inform understanding where FH-specific evidence is limited. Specifically, it evaluates effectiveness in microbiological safety, impact on nutritional and bioactive components, and feasibility for use in low-resource and outbreaks settings.

## 2 Methodology

### 2.1 Protocol

This scoping review followed the Joanna Briggs Institute (JBI) methodology for scoping reviews and adhered to the Preferred Reporting Items for Systematic Reviews and Meta-Analyses Extension for Scoping Reviews (PRISMA-ScR) guidelines (13, 14). The protocol was registered prospectively in Open Science Framework.^1^

To locate the relevant studies, we searched PubMed and Scopus to identify published peer-reviewed literature. The search adhered to the Peer Review of Electronic Search Strategies (PRESS) guidelines to enhance rigor and reduce error (15). The initial search strategy was developed in PubMed and subsequently adapted for Scopus on December 15, 2025. The search strategy was developed collaboratively with support from an informationist at the Welch Medical Library. Following the identification of newly published studies, we expanded our search to five databases: PubMed, Scopus, Web of Science, Embase, and CINAHL. The updated searches across all five databases were conducted on August 30, 2026, to capture the most recent available evidence. Database searches were supplemented through forward citation searching. Forward citation searching included a single-round screening of the reference lists from all included studies and relevant review articles. Non-peer-reviewed evidence (grey literature) was also eligible for inclusion. Grey literature searches were conducted to identify relevant reports, guidance documents, technical briefs, and other materials not published in peer-reviewed journals. Searches were performed on selected organizational and institutional websites (e.g., World Health Organization, PATH) using predefined search terms related to human milk and heat treatment, and where advanced Boolean searching was not available, simplified keyword searches were used. Full list of search terms are listed in Table 1.

**Table 1:** Search terms and strategy.

Source	Search strategy
Peer-reviewed literature	
PubMed	(Pasteurization OR “Flash heating” OR “Flash pasteurization” OR HTST OR “High Temperature Short Time” OR “Pretoria Pasteurization” OR “Heat treatment”) AND (“Milk, Human”[MeSH] OR “Human milk” OR “Donor milk” OR “Breast milk”)
Scopus	TITLE-ABS-KEY(“Human milk” OR “Donor milk” OR “Breast milk”) AND TITLE-ABS-KEY(Pasteurization OR Pasteurisation OR “Flash heating” OR “Flash pasteurization” OR HTST OR “High Temperature Short Time” OR “Pretoria Pasteurization” OR “Heat treatment”)
Embase	#1: ‘human milk’ OR ‘donor milk’ OR ‘breast milk’ #2: ‘pasteurization’ OR ‘pasteurisation’ OR ‘flash heating’ OR ‘flash pasteurization’ OR ‘htst’ OR ‘high temperature short time’ OR ‘pretoria pasteurization’ OR ‘heat treatment’ n#3: #1 AND #2
Web of Science	ALL = (“human milk” OR “donor milk” OR “breast milk”) AND ALL = (pasteurization OR pasteurisation OR “flash heating” OR “flash pasteurization” OR HTST OR “high temperature short time” OR “Pretoria pasteurization” OR “heat treatment”)
CINAHL	(“Human milk” OR “Donor milk” OR “Breast milk”) AND (Pasteurization OR Pasteurisation OR “Flash heating” OR “Flash pasteurization” OR HTST OR “High Temperature Short Time” OR “Pretoria Pasteurization” OR “Heat treatment”)
Grey literature	
World Health Organization (WHO)	(“Human milk” OR “Donor milk” OR “Breast milk”) AND (Pasteurization OR Pasteurisation OR “Flash heating” OR “Flash pasteurization” OR HTST OR “High Temperature Short Time” OR “Pretoria Pasteurization” OR “Heat treatment”)
PATH	“pasteurization”; “flash heating”; “heat treatment breast milk”

### 2.2 Eligibility criteria

Studies were included if they focused on HTST and/or FH of human milk. We included empirical studies (descriptive, experimental, implementation) that report on HTST and/or FH regarding microbiological safety, immunological and nutritional components, and applicability in home-based settings. The search was conducted in English and no restrictions were placed on the date of publication. We excluded studies focusing on non-human milk and non-primary research (i.e., reviews, book chapters, news, poems, biographical items).

### 2.3 Screening and selection process

Two reviewers (HN and CG) screened the title, abstract and keywords of each record identified in the electronic database searches independently. Results from the database searches were imported into Covidence review management software (Veritas Innovation Ltd., Melbourne, Australia) (16). Full-text articles were retrieved for all potentially relevant studies and all studies where the relevance was unclear from screening.

The two reviewers (HN and CG) independently applied the inclusion criteria to each of the studies identified in the searches which passed the title and abstract screening process in order to determine their eligibility for inclusion. Assessment of quality of studies included in the review was not conducted as this scoping review aims to provide an overview of literature. Any disagreements were resolved through consensus building, discussing the study and reaching a conclusion with a third independent reviewer (MV). Identified grey literature was reviewed using the same process. Both peer-reviewed and grey literature were included to map all available evidence on the impact of pasteurization of expressed human milk using FH.

### 2.4 Data extraction and analysis

Two reviewers (HN and CG) independently extracted data from studies identified in the search and screening process as eligible for inclusion in the review. Data was entered into a pre-written data extraction form using a Microsoft Excel spreadsheet including information about the study year of publication, study design, and population setting. For FH, we included technique used, materials/tools, heating time, heating temperature, and type of breastmilk. For pathogen inactivation, pathogen name, detection method, successful inactivation (yes/no) were included. For nutritional integrity, macronutrient retention, micronutrient retention, measurement methods, and reported change were included. For immunological components, immunoglobulin levels, changes reported post heating, and measurement technique used were included. When available, retention values for both nutrients and immunological components were extracted as reported by the original studies. If retention was not reported, it was calculated manually as the post-heating value divided by the pre-heating value and expressed as a percentage. When exact numerical retention values were not reported and were presented only as graphs, values were estimated from the published figures. For additional feasibility, barriers or facilitators reported, cost/availability of materials, safety concerns, and incidents reported were included. For its recommendation, we reviewed if the technique has been included in any technical guideline or policy in the studied country.

The findings were synthesized descriptively and narratively according to pathogen safety, preservation of nutritional and immunological components, and implementation considerations in different emergency contexts in low-resource settings. Studies evaluating HTST identified during the search were used as contextual evidence to provide a broader understanding of the effects of heat treatment of breastmilk. Findings from these studies were not extrapolated to FH because of observed differences in heating duration, temperature, and operational system to conduct the heat treatment technique. These HTST studies were not included in the formal synthesis, are summarised in Supplementary Tables 1, 2, and are briefly discussed narratively where relevant to contextualize the broader evidence on heat treatment of breastmilk.

## 3 Results

A total of 7,030 records were identified from database searches, grey literature, and forward citation searching. After removal of 3,587 duplicates, 3,443 records underwent title and abstract screening. Of 158 full-text articles assessed for eligibility, 24 studies met the inclusion criteria and were included in the review (Figure 1). The included studies (*n* = 24) were published from 2005 to 2025, all reporting findings related to FH (17–40). Study characteristics of the included studies are provided in Supplementary Table 1.

**Figure 1:**
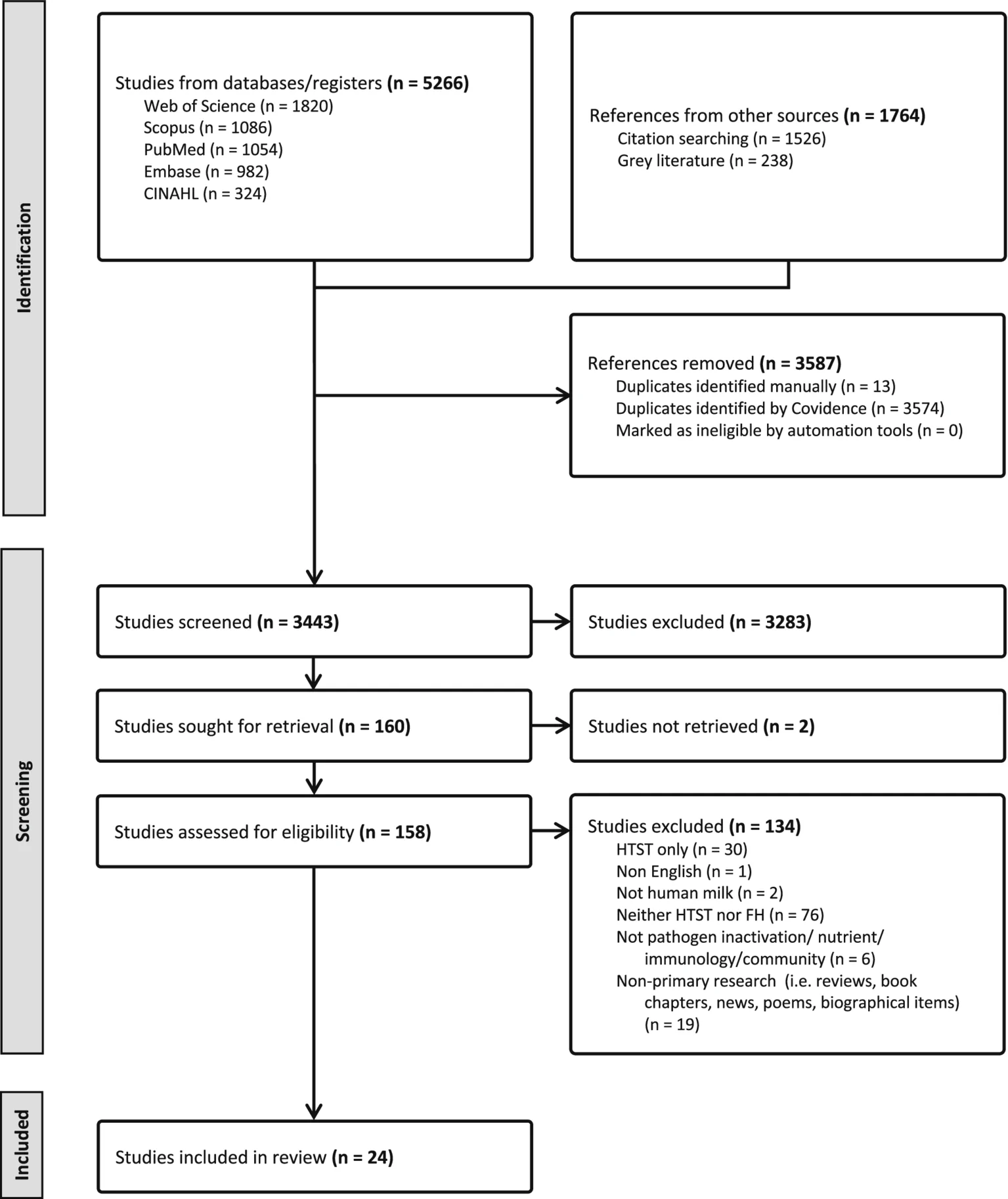
PRISMA flow diagram adapted from Covidence (16).

### 3.1 Microbiological safety following flash-heating of breastmilk

Table 2 summarizes the evidence on FH and bacterial and viral pathogen growth. Existing evidence indicates FH can substantially reduce bacterial contamination. Studies evaluating FH under field and clinical conditions demonstrate reductions in common bacterial contaminants, including *Escherichia coli*, *Staphylococcus aureus*, and Group B *Streptococcus*, organisms which are frequently introduced through handling or maternal mastitis (21, 30, 37). Although *S. aureus* is relatively heat-tolerant, significant reductions in bacterial load have been observed following FH, though complete growth inhibition is not consistently achieved (17, 21).

**Table 2:** Flash-heating effects on pathogen growth inhibition and temperature profiles.

Bacterial pathogen	Bacterial growth observed	Temperature monitoring	Temperature reached (methodology)	Sample size*	Source
Alpha-hemolytic streptococci	No	Yes	72 °C for 30–60 s	100	(37)
*Acinetobacter* spp.	No	Yes	72 °C for 30–60 s	100	(37)
*Bacillus*	No	Yes	Mean peak 79.3 °C	37	(21)
*Brevundimonas diminuta*	No	Yes	72 °C for 30–60 s	100	(37)
*Coliform*	Yes	No	72.9 °C, no time mentioned	10	(22)
*Enterobacter* spp.	No	Yes	72 °C for 30–60 s	100	(37)
*Enterococcus faecalis*	No	Yes	72 °C for 30–60 s	100	(37)
*Escherichia coli*	No	Yes	Mean peak 79.3 °C	37	(21)
	No	Yes	>56 °C for 6 min 15 s; peak 72.9 °C	38	(30)
	No	Yes	72 °C for 30–60 s	100	(37)
	No	No	Not defined	Not reported	(17)
Group B *Streptococcus*	No	Yes	>56 °C for 6 min 15 s; peak 72.9 °C	38	(30)
*Klebsiella* spp.	No	Yes	Mean peak 79.3 °C	37	(21)
	No	Yes	72 °C for 30–60 s	100	(37)
Pseudomonas spp.	No	Yes	72 °C for 30–60 s	100	(37)
*Serratia marcescens*	No	Yes	72 °C for 30–60 s	100	(37)
*Sphingomonas paucimobilis*	No	Yes	72 °C for 30–60 s	100	(37)
*Staphylococcus aureus*	No	Yes	Mean peak 79.3 °C	37	(21)
	No	Yes	>56 °C for 6 min 15 s; peak 72.9 °C	38	(30)
	No	Yes	72 °C for 30–60 s	100	(37)
	No	No	Not defined	Not reported	(17)
*Stenotrophomonas maltophilia*	No	Yes	72 °C for 30–60 s	100	(37)
Non-specified pathogens	No	No	>56 °C for 6 min 15 s; peak 72.9 °C	17	(38)
	No	No	72 °C	30	(23)
	No	Yes	72 °C for 30.7 s	45	(26)

Viral pathogen	Viral growth observed	Temperature monitoring	Temperature reached (methodology)	Sample size*	Source
HIV	No	Yes	>56 °C for 6 min 15 s; peak 72.9°C	98	(31)

*Sample size refers to the flash-heating sample only.

FH has also been shown to reduce viral contamination, specifically HIV viral load, in breastmilk, including cell-free and cell-associated virus (31). These findings are consistent with the known heat sensitivity of other lipid-enveloped viruses (41).

Additional evidence of virus susceptibility comes from laboratory HTST studies, which demonstrate that most vegetative bacteria and enveloped viruses are readily inhibited at temperatures around 72 °C (42, 43). In contrast, non-enveloped viruses such as porcine parvovirus show marked heat resistance, suggesting that similar pathogens may be less susceptible to growth inhibition by brief heat exposure (see Supplementary Table 1). An additional study found that flash-heated samples showed significantly reduced levels of non-pathogenic bacterial growth over an 8-h period at room temperature, compared to unheated breastmilk (30). Overall, available evidence suggests that FH is effective in reducing bacterial contamination and inactivating or reducing viral load in heat-sensitive viruses in human milk.

Temperatures achieved varied between studies. Additionally, in 50% of the studies investigating FH and pathogen inactivation, the temperature of breast milk was not directly measured; however, the methods stated that the milk was assumed to have reached 72 °C for 15 s following application of the FH protocol.

### 3.2 Nutritional integrity following flash-heating of breastmilk

The impact of FH on nutritional components of human milk is shown in Table 3. FH had no negative impact on the total carbohydrates and fat content of milk (38, 39). Similarly, micronutrients (vitamin A, vitamin B1, vitamin B6, vitamin B12, vitamin E) were well preserved with little to no negative impact (28, 32, 38). Vitamin B2 retention ranged from 59 to 100%. Vitamin C and folate retention varied depending on the specific FH procedure, ranging from 39 to 100% for vitamin C and 76–100% for folate (28, 32, 38). The total oligosaccharides (the largest sugar component) were completely preserved (24). The lipase activity (important for fat digestion in infants) was, as expected, almost completely destroyed by heat treatment (34, 38).

**Table 3:** Flash-heating effects on retention (%) of immunological and nutrient components.

Components	FH without monitoring of milk temperature	FH with temperature monitoring—thermometer in 50 mL of breastmilk samples	FH-temperature monitored in 120 mL of breastmilk samples
Retention of immunological components			
Interleukin−8 (IL-8) concentration			100% (25)
IL-10 concentration			97% (25)
Total IgA concentration	60% (34)	80% (19)	25% (25); 34% (26)
Total IgG concentration		67% (19)	
Lactoferrin concentration	26% (38); 30% (34)	16% (28)	
Lysozyme concentration	60% (34)	21% (28)	
Lactoferrin bactericidal activity		89% (20)	39% (25); 40% (26)
Lysozyme bactericidal activity	56% (38)	43% (20)	78% (25)
Retention of nutrient components			
Total oligosaccharides			100% (24)
Carbohydrates and fats	100% (38, 39)		
Vitamin A		100%^a^ (28); 94% (32)	
Vitamin B1		100%^a^ (28)	
Vitamin B2		100%^a^ (28); 59% (32)	
Vitamin B6		100%^a^ (28); 96% (32)	
Folate	76% (38)	100%^a^ (28); 100%^a^ (32)	
Vitamin B12		100%^a^ (28); 100%^a^ (32)	
Vitamin C	39% (38)	79% (28); 100%^a^ (32)	
Vitamin E		90% (28)	
Lipase activity	<1% (38); <10% (34)		

a Values initially exceeded 100% and are reported as ~100%, reflecting post-heating increases, which may be attributable to the release of nutrients from binding proteins during heating.

Francisco et al. (27) did not report percentage retention of IgA and therefore are not represented in the table; however, they reported no statistically significant reduction in IgA following flash-heating.

The impact of FH on the preservation of other nutritional components can be inferred from HTST, which has been studied in depth (see Supplementary Table 2). Total antioxidant capacity and total protein were generally well preserved under HTST (43, 44). Bile salt-stimulated lipase (BSSL), which is important for fat digestion in infants, was largely inactivated by HTST treatment (34, 43, 45, 46). Fat composition was largely preserved following HTST, with longer treatment durations (20–25 s) were associated with reduced saturated fatty acids and relatively increased polyunsaturated fatty acids (47).

Overall, it appears that FH of human milk preserves many nutritional components, but can substantially reduce the activity of certain bioactive proteins and enzymes, particularly lipase. The extent of these changes is dependent on the temperature achieved and duration of heating.

### 3.3 Immunological preservation following flash-heating of breastmilk

Immunoglobulins (IgA and IgG) were found only to be preserved to a moderate degree (25–80%) following FH as shown in Table 3, although the level of preservation varied by peak temperature and volume of milk heated. Cytokines (IL-8 and IL-10) were well preserved after FH, with retention approaching 100% (25). In contrast, lactoferrin and lysozyme concentrations showed lower retention, ranging from 16 to 30% (28, 38), and 21–60% (34) respectively. The bactericidal activity of both lactoferrin and lysozyme was relatively better preserved. Lactoferrin activity retention ranged from 38.6 to 89% (20, 25, 26). A similar wide range in retention was seen for lysozyme bactericidal activity, 43–78.4% (20, 25, 38).

Evidence from FH studies showed broadly consistent findings in the preservation of immunological components as those observed in HTST studies, with some differences (see Supplementary Table 2). In HTST, immunoglobulins were generally preserved to a moderate degree, with IgA consistently better preserved than IgG and IgM. The highest retention was observed under HTST conditions of 62 °C for 5 s (46). Retention declined with increasing temperatures and longer heating duration (34, 43, 46, 48). IgG and IgM were typically retained at approximately 50–80% under HTST with temperatures ranging from 71 to 73 °C and durations of 5–9 s (48). Evidence on lysozyme under HTST differed from that observed under FH (reduction on the concentration) with studies indicating its heat-resistant properties and no significant reduction following HTST (43, 49). In contrast, lactoferrin levels showed heat sensitivity, with retention of approximately 30–50% under HTST (34, 46).

Overall, FH of breastmilk resulted in a greater reduction of immune components when compared to HTST. Nevertheless, at least one-third of most immunological components, including immunoglobulins and cytokines, remain preserved, although lactoferrin concentration was substantially reduced.

Findings on the impact on immunological components from FH performed in home settings without temperature monitoring are limited (only 2 out of 7 studies) (34, 38). This highlights an important research gap regarding how analyte concentrations could change over time when exposed to heat.

### 3.4 Feasibility of flash-heating in household settings

The feasibility of FH in household settings has been examined across several domains, including community awareness and acceptability of heat treatment of breastmilk, behavioral uptake among HIV-infected mothers, barriers and facilitators to adoption.

#### 3.4.1 Community awareness and acceptability of heat treatment

Community awareness and acceptability of heat treatment of breastmilk has only been researched in relation to HIV. Results varied across settings. A qualitative study in three Zimbabwe regions among mothers and community members reported that heat treatment of expressed breastmilk was initially met with skepticism due to concerns about the safety and potential nutrient loss (29). However, after discussion and information sharing, acceptance of heat treatment as a feasible infant-feeding option improved. Low cost, convenience, and consistent availability of breastfeeding compared with alternative feeding options, such as CMF or other BMS were reported as reasons for acceptability (29). In a community-based intervention in a rural region of Zimbabwe, awareness of heat treatment for breastmilk was high and increased further among participants exposed to community health promotion activities using multichannel media (33). In contrast, initial formative research in Tanzania documenting beliefs associated with a range of HIV prevention interventions showed strong community resistance to both expression and heat treatment of breastmilk (35). These practices were considered as time-consuming and culturally unfamiliar; notably, these perceptions were voiced in the absence of discussion and open dialogue on the topic (35).

#### 3.4.2 Behavioral uptake of flash-heating

Behavioral uptake of FH practices in the context of HIV was generally favorable across settings. Data collected from home-based infant feeding counseling among HIV-infected mothers in Tanzania showed that 51.4% initiated FH over the subsequent 6 months. Uptake did not vary by age, religion, parity, or education among those who practiced FH, and mothers practiced FH for an average of 9.6 weeks (21). Similarly, 20 HIV-infected mothers in resource-poor rural Zimbabwe adopted FH and practiced it for a mean duration of 4.5 months with home-based support from nurses (36). Mothers who practiced FH were generally able to manually express their milk and performed procedures correctly according to the recommended protocol in the study, including cleaning utensils, washing hands, and using the correct amount of water for heating. Peak milk temperatures achieved during heating were 79.3–80.5 °C (21, 36). FH was implemented using readily available household materials, including glass jars, aluminum pans, cups, and commonly used fuels such as kerosene and charcoal. Heated water was also often reused for cooking or bathing (21). Another study among HIV-infected mothers in South Africa reported high uptake of FH at home among mothers who believed FH would prevent HIV transmission, and that they could express breastmilk for 4 months and easily heat treat it (18).

#### 3.4.3 Barriers and facilitators to flash-heating adoption

Qualitative insights from HIV-infected mothers in Tanzania identified barriers and facilitators influencing the adoption of FH. Reported barriers included difficulties with expressing breastmilk, shortage of fuel for heating, conflicting guidance from health workers, and limited familiarity with heating practices (40). Key facilitators included peer counselor support, household support from husbands, low-cost materials, and perceived benefits for infant health (40). Providing counseling support, basic equipment, and social marketing were also reported as important factors to address barriers to FH and enable adoption of FH (36).

## 4 Discussion

This scoping review included 24 studies published between 2005 and 2025 across 7 countries: South Africa (*n* = 12), Tanzania (*n* = 3), Zimbabwe (*n* = 3), Canada (*n* = 2), Indonesia (*n* = 1), the United States of America (*n* = 1), the Netherlands (*n* = 1). One study did not report the country in which the study was conducted (27). The available evidence suggests that FH may inhibit growth a range of pathogens while preserving substantial proportions of key nutritional and immunological components of human milk. Available studies further indicate the potential feasibility of FH in low-resource settings. However, evidence remains limited and heterogeneous between studies. This review outlines the need for standardized FH protocols, outcome measures, and reporting practices to improve comparability across studies.

### 4.1 Role and importance of breastfeeding

Breastfeeding should be preserved in disease epidemics wherever possible. Breastfeeding is a foundation for the health of both mothers and children (50, 51). It protects against childhood infections, hospitalizations, mortality, allergies, cardiovascular disease, and chronic conditions such as obesity and diabetes, in addition to enabling normal growth and neurodevelopment (52–54). On the mothers’ side it supports sensitive, responsive care and protects against maternal maltreatment, while offering benefits for maternal health, including postpartum recovery, mental health, enhanced metabolic health, and lower risks of ovarian and breast cancer (51, 52). Maintaining milk expression in the absence of direct breastfeeding can support maternal confidence and build emotional connection with the infant (51, 55, 56). Breastfeeding also carries significant economic implications, as its premature cessation translates into an increased expenditure on alternative feeding options- such as infant formula- and healthcare costs resulting from higher incidence of illness (57, 58).

Global guidance for infant and young child feeding establishes a contextual hierarchy for situations when breastfeeding is disrupted, namely, mother’s own expressed milk and when that is not available then the milk of a healthy wet-nurse, then donor human milk from a human milk bank, with fed by a cup as the last resort (59). In emergencies, where an infant is not breastfed, the Operational Guidance for Infant and Young Child Feeding in Emergencies reinforces the importance of exploring breastmilk options before considering CMF or other BMS (60). CMF feeding poses serious challenges related to lack of safety, availability and affordability, whereas breastmilk is a critical, locally available and sustainable food source, particularly during emergencies (61). As described, breastmilk holds intrinsic value beyond its nutritional and immunological properties, including psychological, relational, and societal benefits (62); discarding it represents the loss of a valuable resource. As such, when public health emergencies and infectious disease outbreaks interfere with breastfeeding and lead to mother–infant separation, feeding expressed maternal milk remains important to be explored, including with heat treatment for pathogen elimination (63).

### 4.2 Flash-heating effects on pathogens present in breastmilk

Based on our findings, FH is a promising strategy in situations where disease transmission via breastmilk is a serious concern. FH has been shown to inhibit growth of a range of pathogens while preserving substantial proportions of key nutrients and immunological components in human milk. These findings suggest that FH may offer a practical compromise, between ensuring microbiological safety and maintaining the health benefits of breastfeeding and food security for infants in outbreak settings. It presents an option for risk-reduction where artificial feeding is not feasible or is dangerous.

The effectiveness of FH in pathogen growth has been demonstrated across multiple studies, with evidence of both partial reduction and complete inhibition depending on the micro-organism. While bacterial pathogens have been the primary focus, viral pathogens have also been investigated, with varying levels of susceptibility to heat. In general, many pathogens growth is inhibited at temperatures above 60 °C, which are achievable through FH methods (64–68). However, differences in thermal resistance between pathogens may explain why some viruses’ growth is more inhibited than others. Importantly, the current evidence base is limited by the range of pathogens studied in the application of FH, highlighting the need for pathogen-specific evaluations as well as further research required for other pathogens that can cause outbreaks such as Ebola virus, CMV, Zika virus, among others. Additionally, contextual factors such as altitude and heating rates may influence the temperatures achieved, thereby affecting pathogen growth (69).

Another consideration when applying thermal treatments is the preservation of the immunological and nutritional properties of breastmilk. In this review, some components were found to be affected by FH, including reductions in concentrations of immunological factors such as lysozyme, as well as certain vitamins, including vitamins E and C. In contrast, some studies reported increases in levels of other nutrients, such as B vitamins and folate, although these findings may be influenced by methodological factors (38). Overall, it is important to recognize that breastmilk retains a wide range of bioactive components even after heat treatment that are not present in BMS (70).

### 4.3 Impact of flash-heating on immunological and nutritional components

The immunological composition of breastmilk is not static and varies across the postpartum period (8, 71). As a result, the impact of FH on immune components may differ depending on the stage of lactogenesis. Nevertheless, even with partial degradation of immunological factors, heat-treated breastmilk will remain nutritionally and biologically superior to BMS. BMS, including CMF, lack critical immune and bioactive components and cannot replicate the protective functions of breastmilk, including immune system activation and metabolic programming (70, 72, 73). Additionally, BMS does not contain the hundreds of oligosaccharides and prebiotic sugars that are in breastmilk, which support growth of the commensal bacteria in the infant’s gut, and amongst other things aid in production of short-chain fatty acids necessary for healthy infant brain development (70). Furthermore, oligosaccharides, which are preserved after FH, also function as immunological components, inhibiting pathogen adhesion and modulating immune responses (74). This is of clinical significance because human milk oligosaccharides provide the food source for the commensal bacteria of the infant’s microbiome, thus inhibiting pathogen adhesion and modulating immune responses (74). This could provide some compensation for the other components impacted during FH. Finally, CMF and other BMS make infants susceptible to colonisation with pathogens via disruption of the intestinal mucosa and other alterations to the intestinal environment (75).

### 4.4 Standardization challenges in flash-heating research

Overall, FH shows promise as an accessible and low-cost intervention in emergency settings, and further research is needed to standardize methodologies and validate its effectiveness under real-world conditions. Methodological variability across studies limits the comparability and reproducibility of existing evidence. A key challenge is the lack of standardization regarding monitoring the temperature of milk during FH. Although many studies report targeting temperatures around 72 °C, actual temperatures are not always directly measured or consistently verified. Indeed, the FH methodology was developed for in-home use where thermometers were not necessarily available. Variations in experimental setup including water volume, container type, use of lids, and heating duration contribute to potential inconsistencies in temperature achieved via FH. FH with temperature monitoring of milk facilitated by the FoneAstra or PiAstra device has been shown to be effective and feasible (24, 26). However, with FH, where the milk temperature was not monitored, the effects on immunological and nutritional components has not been adequately evaluated, and variability in achieved temperatures could affect the preservation of these components.

### 4.5 Feasibility and acceptability of flash-heating

While FH has been explored in household contexts in non-emergency settings, its practicality during outbreaks requires further examination. Reported barriers to FH adoption underscore the need for practical guidance, education, and careful consideration of local resources (fuel and equipment). The research on acceptability and uptake of FH shows that women’s motivation to protect their infants, including via expression and heat treatment of their breastmilk, should not be discounted or overlooked. Other feeding methods such as use of CMF, also require substantial work and resources for preparation and storage, including fuel, water, formula, and utensils, which is a critical reminder that resource demands are not unique to FH (76). These feasibility and acceptability considerations also point towards the broader implications at the policy and implementation level. Greater integration and alignment across nutrition, emergency response, infectious disease, and humanitarian systems may be needed to support consistent and coordinated guidance. Although existing frameworks such as the global Operational Guidance on Infant and Young Child Feeding in Emergencies (OG-IFE) provide an important foundation, gaps remain in their operationalization when breastfeeding is interrupted by outbreaks (60). In practice, there is a lack of clear context-specific recommendations to support decision-making where competing risks must be weighted, including limited direction on safely maintaining a human milk diet during separation or when maternally expressed milk could be a transmission risk. However, as Rollins et al. (77) highlights that infant feeding policies should not be solely based on the risk of pathogen transmission, but balancing the risk against well-established consequences of disrupting breastfeeding and mother-infant contact, including impacts on child survival, health, development, and maternal well-being. Strengthening the integration of feasible low-resource strategies like heat treatment of expressed breastmilk could support continuity of human milk feeding in low-resource, emergency settings. More generally, there needs to be greater investment in research on breastfeeding and breastmilk feeding in infectious disease epidemics (78).

### 4.6 Strengths and limitations

The absence of a formal quality or risk-of-bias assessment limits the ability to evaluate the methodological robustness of the included evidence. In addition, the included studies varied considerably in study design, sample size, experimental conditions, and reporting of operational procedures. Not all studies provided complete methodological details, and therefore only the information reported in the published articles could be extracted and evaluated. This heterogeneity may limit the comparability and interpretation of findings across studies. As the first review to examine FH in the context of potential application during disease outbreaks, this study provides an overview of the nature, scope, and characteristics of the existing evidence base, while identifying important methodological and knowledge gaps that should be addressed in future research.

## 5 Conclusion

In conclusion, flash-heating shows potential as an alternative option to support breastmilk feeding when direct breastfeeding and/or use of untreated breastmilk is considered not safe. In emergencies, it is critical to recognize that the choice is not between flash-heating and a risk-free alternative, it is between flash-heating and the known, well-documented risks of CMF and other BMS in low-resource settings. Given the evidence, it appears that in many circumstances FH may represent a safer and more feasible option for preserving the value of breastmilk. Further research is needed to explore its applicability and adaptability to outbreak settings, including challenges related to standardization linked to the procedures, feasibility, and acceptability.

### 5.1 Future direction

Future studies should evaluate the impact of FH on a broader range of pathogens and in other infectious disease outbreak-prone settings beyond those represented in this current evidence base, while also establishing standardized and reproducible protocols for its implementation. This includes defining clear parameters for heating duration, target temperature, and acceptable variability, supported by simple, low-cost tools such as thermometers placed directly in the milk or other calibrated household devices that can reliably indicate when the target temperature has been reached as well as provide tracking of temperatures for continual monitoring and quality assurance. Evaluating the feasibility, accuracy, and usability of these tools in outbreak settings will be essential to ensure consistent application and adherence to safety protocols.

Ensuring safety extends beyond temperature monitoring alone, including the risk of contamination post-heat treatment. Future research should examine the full operational pathway of FH, including safe milk expression, heating procedures, milk collection and storage containers, heat sources, post-heating handling, storage conditions, feeding practices and data tracking. In particular, the risk of recontamination following heat treatment remains an important gap in the current evidence and should be evaluated across these steps to better understand how this risk may be minimized in household settings. These components should be evaluated as an integrated system under real-world constraints to determine safety and feasibility in practice.

Importantly, future research should also assess the feasibility, acceptability, and willingness of mothers, families, and frontline health workers to adopt FH in outbreak settings. This includes evaluating ease of use, time burden, cultural acceptability, and perceived risks and safety of the method. Engaging these stakeholders not only as participants but as active contributors in shaping research questions and implementation approaches will be critical to ensure that strategies are grounded in lived realities rather than perceived notions about what is achievable.

Strengthening this comprehensive evidence base will be essential to support clear, context-sensitive public health recommendations and operational guidance for the safe and feasible use of FH as an human milk feeding strategy in outbreak settings. Beyond the operational aspects of FH, important gaps to be addressed in future research remain regarding its long-term safety and clinical outcomes, including infection-related outcomes, infant growth and development, and potential effects on the gut microbiota.

## Footnotes

1
https://doi.org/10.17605/OSF.IO/P6S4A
