# Supplementary material for: A scoping review of microbiological safety, bioactive components, nutrient retention, and feasibility of flash-heating expressed breastmilk: exploring potential use during infectious disease outbreaks
Source: Front Nutr. 2026 Oct 15;13:1926806. doi: 10.3389/fnut.2026.1926806 (PMC13717084; doi:10.3389/fnut.2026.1926806)
Supplement: Supplementary file 1 [file Table_1.docx]

**Supplementary Material**

**Supplementary Table 1. Study characteristics**

| **Author, Publication Year** | **Country** | **Study design** | **Sample size*** | **References** |
| --- | --- | --- | --- | --- |
| Besser, 2013 | South Africa | Experimental | NR | 17 |
| Chaponda, 2016 | South Africa | Cross-sectional | 70 | 18 |
| Chantry, 2009 | South Africa | Experimental | 50 | 19 |
| Chantry, 2011 | South Africa | Experimental | 50 | 20 |
| Chantry, 2012 | Tanzania | Longitudinal cohort | 101 | 21 |
| Choiriyah, 2025 | Indonesia | Experimental | 10 | 22 |
| Coutsoudis, 2011 | South Africa | Longitudinal cohort | 191 | 23 |
| Daniels, 2017 | South Africa | Experimental | 48 | 24 |
| Daniels, 2017 | South Africa | Experimental | 50 | 25 |
| Daniels, 2018 | South Africa | Experimental | 45 | 26 |
| Fransisco, 2018 | NR | Experimental | 20 | 27 |
| Israel-Ballard, 2005 | USA | Experimental | 10 | 28 |
| Israel-Ballard, 2006 | Zimbabwe | Qualitative | 77 | 29 |
| Israel-Ballard, 2006 | South Africa | Experimental | 38 | 30 |
| Israel-Ballard, 2007 | South Africa | Experimental | 84 | 31 |
| Israel-Ballard, 2008 | South Africa | Experimental | 50 | 32 |
| Jenkins, 2012 | Zimbabwe | Cross-sectional | 462 | 33 |
| Kontopodi, 2022 | Netherlands | Experimental | 4 | 34 |
| Leshabari, 2006 | Tanzania | Qualitative | 15 participants and 8 focus groups with 8-12 participants each | 35 |
| Mbuya, 2010 | Zimbabwe | Mixed-methods | 20 | 36 |
| Naicker, 2015 | South Africa | Experimental | 100 | 37 |
| Pitino, 2019 | Canada | Experimental | 17 | 38 |
| Pitino, 2019 | Canada | Experimental | 8 | 39 |
| Young, 2013 | Tanzania | Qualitative | 144 | 40 |
| *Sample size refers to the total study population reported by the authors, rather than the number of participants in a specific subgroup, intervention, or analysis. Where sample size varies across variables or assays, the largest reported sample size is presented.  **NR**: Not reported in the study. | | | | |
