# Supplementary material for: A scoping review of microbiological safety, bioactive components, nutrient retention, and feasibility of flash-heating expressed breastmilk: exploring potential use during infectious disease outbreaks
Source: Front Nutr. 2026 Oct 15;13:1926806. doi: 10.3389/fnut.2026.1926806 (PMC13717084; doi:10.3389/fnut.2026.1926806)
Supplement: Supplementary file 2 [file Table_2.docx]

**Supplementary Material**

**Supplementary Table 2. High Temperature Short Time (HTST) Effects on Pathogen Growth Inhibition and Temperature Profiles**

| **Bacterial Pathogen** | **Bacterial Growth Observed** | **Temperature Monitoring** | **Temperature Reached (Methodology)** | **Source** |
| --- | --- | --- | --- | --- |
| *Acinetobacter baumanni complex* | No | Yes | 70 °C for 5 s; 70 °C for 10 s; 70 °C for 15 s; 70 °C for 20 s; 70 °C for 25 s; 72 °C for 5 s; 72 °C for 10 s; 72 °C for 15 s; 72 °C for 20 s; 72 °C for 25 s; 75 °C for 5 s; 75 °C for 10 s; 75 °C for 15 s; 75 °C for 20 s; 75 °C for 25 s | (1) |
| *Acinetobacter ursingii* | No | Yes | 70 °C for 5 s; 70 °C for 10 s; 70 °C for 15 s; 70 °C for 20 s; 70 °C for 25 s; 72 °C for 5 s; 72 °C for 10 s; 72 °C for 15 s; 72 °C for 20 s; 72 °C for 25 s; 75 °C for 5 s; 75 °C for 10 s; 75 °C for 15 s; 75 °C for 20 s; 75 °C for 25 s | (1) |
| *Bacillus cereus* | Yes | Yes | 70 °C for 5 s; 70 °C for 10 s; 70 °C for 15 s; 70 °C for 20 s; 70 °C for 25 s; 72 °C for 5 s; 72 °C for 10 s; 72 °C for 15 s; 72 °C for 20 s; 72 °C for 25 s; 75 °C for 5 s; 75 °C for 10 s; 75 °C for 15 s; 75 °C for 20 s; 75 °C for 25 s | (1) |
| *Chryseobacterium indologenes* | No | Yes | 70 °C for 5 s; 70 °C for 10 s; 70 °C for 15 s; 70 °C for 20 s; 70 °C for 25 s; 72 °C for 5 s; 72 °C for 10 s; 72 °C for 15 s; 72 °C for 20 s; 72 °C for 25 s; 75 °C for 5 s; 75 °C for 10 s; 75 °C for 15 s; 75 °C for 20 s; 75 °C for 25 s | (1) |
| *Clostridium ramosum* | No | Yes | 70 °C for 5 s; 70 °C for 10 s; 70 °C for 15 s; 70 °C for 20 s; 70 °C for 25 s; 72 °C for 5 s; 72 °C for 10 s; 72 °C for 15 s; 72 °C for 20 s; 72 °C for 25 s; 75 °C for 5 s; 75 °C for 10 s; 75 °C for 15 s; 75 °C for 20 s; 75 °C for 25 s | (1) |
| *Coagulase-negative staphylococci* | No | Yes | 62 °C for 5 s | (2) |
| *Corynebacterium tuberculostearicum* | No | Yes | 70 °C for 5 s; 70 °C for 10 s; 70 °C for 15 s; 70 °C for 20 s; 70 °C for 25 s; 72 °C for 5 s; 72 °C for 10 s; 72 °C for 15 s; 72 °C for 20 s; 72 °C for 25 s; 75 °C for 5 s; 75 °C for 10 s; 75 °C for 15 s; 75 °C for 20 s; 75 °C for 25 s | (1) |
| *Cronobacter sakazakii* | No | Yes | 72 °C for 15 s | (3) |
| *Cronobacter sakazakii* | Yes | Yes | 62.5 °C for 5 s | (4) |
| *Cronobacter sakazakii* | No | Yes | 81 °C for 5 s | (4) |
| *Enterobacter asburiae/cloacae* | No | Yes | 70 °C for 5 s; 70 °C for 10 s; 70 °C for 15 s; 70 °C for 20 s; 70 °C for 25 s; 72 °C for 5 s; 72 °C for 10 s; 72 °C for 15 s; 72 °C for 20 s; 72 °C for 25 s; 75 °C for 5 s; 75 °C for 10 s; 75 °C for 15 s; 75 °C for 20 s; 75 °C for 25 s | (1) |
| *Enterococcus faecalis* | Yes, reduced but not eliminated | Yes | 62 °C for 5 s; 62 °C for 15 s; 72 °C for 5 s; 72 °C for 15 s | (5) |
|  | No | Yes | 87 °C for 2 s; 87 °C for 5 s | (5) |
|  | No | Yes | 72 °C for 10 s; 72 °C for 15 s; 72 °C for 20 s; 72 °C for 25 s; 75 °C for 5 s; 75 °C for 10 s; 75 °C for 15 s; 75 °C for 20 s; 75 °C for 25 s | (1) |
|  | Yes | Yes | 70 °C for 5 s; 70 °C for 10 s; 70 °C for 15 s; 70 °C for 20 s; 70 °C for 25 s; 72 °C for 5 s |  |
|  | Yes, reduced but not eliminated (greater reduction at 81°C/5 s) | Yes | 62.5 °C for 5 s; 81 °C for 5 s | (4) |
| *Enterobacter gergoviae* | No | Yes | 70 °C for 5 s; 70 °C for 10 s; 70 °C for 15 s; 70 °C for 20 s; 70 °C for 25 s; 72 °C for 5 s; 72 °C for 10 s; 72 °C for 15 s; 72 °C for 20 s; 72 °C for 25 s; 75 °C for 5 s; 75 °C for 10 s; 75 °C for 15 s; 75 °C for 20 s; 75 °C for 25 s | (1) |
| *Enterobacteriaceae* | No | Yes | 72 °C for 15 s | (6) |
| *Enterococcus spp.* | No | Yes | 62 °C for 5 s | (2) |
| *Escherichia coli* | Yes, at 18.5 s | Yes | 71 °C for 18.5 s; 71 °C for 9 s; 71 °C for 5.75 s | (7) |
|  | No | Yes | 64 °C for 33 s | (8) |
|  | No | Yes | 75 °C for 15 s | (9) |
|  | No | Yes | 72 °C for 16 s | (10) |
| *Gram-negative bacteria* | No | Yes | 62 °C for 5 s | (2) |
| *Klebsiella oxytoca* | No | Yes | 70 °C for 5 s; 70 °C for 10 s; 70 °C for 15 s; 70 °C for 20 s; 70 °C for 25 s; 72 °C for 5 s; 72 °C for 10 s; 72 °C for 15 s; 72 °C for 20 s; 72 °C for 25 s; 75 °C for 5 s; 75 °C for 10 s; 75 °C for 15 s; 75 °C for 20 s; 75 °C for 25 s | (1) |
| *Klebsiella pneumoniae* | No | Yes | 70 °C for 5 s; 70 °C for 10 s; 70 °C for 15 s; 70 °C for 20 s; 70 °C for 25 s; 72 °C for 5 s; 72 °C for 10 s; 72 °C for 15 s; 72 °C for 20 s; 72 °C for 25 s; 75 °C for 5 s; 75 °C for 10 s; 75 °C for 15 s; 75 °C for 20 s; 75 °C for 25 s | (1) |
|  | No (except for 62 °C for 15 s) | Yes | 62 °C for 5 s; 62 °C for 15 s; 72 °C for 5 s; 72 °C for 15 s; 87 °C for 2 s; 87 °C for 5 s | (5) |
| *Klebsiella pneumoniae* | No | Yes | 64 °C for 33 s | (8) |
| *Listeria monocytogenes* | No | Yes | 72 °C for 15 s | (3) |
| *Listeria monocytogenes (strain 00218)* | Yes, reduced but not eliminated (greater reduction at 81°C/5 s) | Yes | 62.5 °C for 5 s; 81 °C for 5 s | (4) |
| *Listeria monocytogenes (strain 0015)* | Yes | Yes | 62.5 °C for 5 s | (4) |
| *Listeria monocytogenes (strain 0015)* | No | Yes | 81 °C for 5 s | (4) |
| *Pantoea aglomerans* | No | Yes | 70 °C for 5 s; 70 °C for 10 s; 70 °C for 15 s; 70 °C for 20 s; 70 °C for 25 s; 72 °C for 5 s; 72 °C for 10 s; 72 °C for 15 s; 72 °C for 20 s; 72 °C for 25 s; 75 °C for 5 s; 75 °C for 10 s; 75 °C for 15 s; 75 °C for 20 s; 75 °C for 25 s | (1) |
| *Pseudomonas aeruginosa* | No | Yes | 70 °C for 5 s; 70 °C for 10 s; 70 °C for 15 s; 70 °C for 20 s; 70 °C for 25 s; 72 °C for 5 s; 72 °C for 10 s; 72 °C for 15 s; 72 °C for 20 s; 72 °C for 25 s; 75 °C for 5 s; 75 °C for 10 s; 75 °C for 15 s; 75 °C for 20 s; 75 °C for 25 s | (1) |
|  | Yes, reduced but not eliminated | Yes | 62 °C for 5 s; 62 °C for 15 s | (5) |
|  | No | Yes | 72 °C for 5 s; 72 °C for 15 s; 87 °C for 2 s; 87 °C for 5 s | (5) |
| *Pseudomonas fluorescens* | No | Yes | 70 °C for 5 s; 70 °C for 10 s; 70 °C for 15 s; 70 °C for 20 s; 70 °C for 25 s; 72 °C for 5 s; 72 °C for 10 s; 72 °C for 15 s; 72 °C for 20 s; 72 °C for 25 s; 75 °C for 5 s; 75 °C for 10 s; 75 °C for 15 s; 75 °C for 20 s; 75 °C for 25 s | (1) |
| *Pseudomonas putida* | No | Yes | 70 °C for 5 s; 70 °C for 10 s; 70 °C for 15 s; 70 °C for 20 s; 70 °C for 25 s; 72 °C for 5 s; 72 °C for 10 s; 72 °C for 15 s; 72 °C for 20 s; 72 °C for 25 s; 75 °C for 5 s; 75 °C for 10 s; 75 °C for 15 s; 75 °C for 20 s; 75 °C for 25 s | (1) |
| *Serratia grimessi* | No | Yes | 70 °C for 5 s; 70 °C for 10 s; 70 °C for 15 s; 70 °C for 20 s; 70 °C for 25 s; 72 °C for 5 s; 72 °C for 10 s; 72 °C for 15 s; 72 °C for 20 s; 72 °C for 25 s; 75 °C for 5 s; 75 °C for 10 s; 75 °C for 15 s; 75 °C for 20 s; 75 °C for 25 s | (1) |
| *Serratia liquefaciens* | No | Yes | 70 °C for 5 s; 70 °C for 10 s; 70 °C for 15 s; 70 °C for 20 s; 70 °C for 25 s; 72 °C for 5 s; 72 °C for 10 s; 72 °C for 15 s; 72 °C for 20 s; 72 °C for 25 s; 75 °C for 5 s; 75 °C for 10 s; 75 °C for 15 s; 75 °C for 20 s; 75 °C for 25 s | (1) |
| *Serratia marcescens* | No | Yes | 70 °C for 5 s; 70 °C for 10 s; 70 °C for 15 s; 70 °C for 20 s; 70 °C for 25 s; 72 °C for 5 s; 72 °C for 10 s; 72 °C for 15 s; 72 °C for 20 s; 72 °C for 25 s; 75 °C for 5 s; 75 °C for 10 s; 75 °C for 15 s; 75 °C for 20 s; 75 °C for 25 s | (1) |
|  | No (except for 62 °C for 5 s and 62 °C for 15 s) | Yes | 62 °C for 5 s; 62 °C for 15 s; 72 °C for 5 s; 72 °C for 15 s; 87 °C for 2 s; 87 °C for 5 s | (5) |
| *Staphylococcus aureus* | Yes, at 18.5 s | Yes | 71 °C for 18.5 s; 71 °C for 9 s; 71 °C for 5.75 s | (7) |
|  | No | Yes | 72 °C for 15 s | (6) |
|  | No | Yes | 72 °C for 16 s | (10) |
|  | No | Yes | 72 °C for 15 s | (3) |
|  | Yes, reduced but not eliminated | Yes | 62 °C for 5 s; 62 °C for 15 s; 72 °C for 5 s; 72 °C for 15 s | (5) |
|  | No | Yes | 87 °C for 2 s; 87 °C for 5 s | (5) |
|  | No | Yes | 70 °C for 5 s; 70 °C for 10 s; 70 °C for 15 s; 70 °C for 20 s; 70 °C for 25 s; 72 °C for 5 s; 72 °C for 10 s; 72 °C for 15 s; 72 °C for 20 s; 72 °C for 25 s; 75 °C for 5 s; 75 °C for 10 s; 75 °C for 15 s; 75 °C for 20 s; 75 °C for 25 s | (1) |
| *Staphylococcus epidermidis* | No | Yes | 70 °C for 5 s; 70 °C for 10 s; 70 °C for 15 s; 70 °C for 20 s; 70 °C for 25 s; 72 °C for 5 s; 72 °C for 10 s; 72 °C for 15 s; 72 °C for 20 s; 72 °C for 25 s; 75 °C for 5 s; 75 °C for 10 s; 75 °C for 15 s; 75 °C for 20 s; 75 °C for 25 s | (1) |
| *Stenotrophomonas maltophilia* | No | Yes | 70 °C for 5 s; 70 °C for 10 s; 70 °C for 15 s; 70 °C for 20 s; 70 °C for 25 s; 72 °C for 5 s; 72 °C for 10 s; 72 °C for 15 s; 72 °C for 20 s; 72 °C for 25 s; 75 °C for 5 s; 75 °C for 10 s; 75 °C for 15 s; 75 °C for 20 s; 75 °C for 25 s | (1) |
| *Streptococcus agalactiae* | No | Yes | 72 °C for 16 s | (10) |
| *Staphylococcus lugdunensis* | No | Yes | 70 °C for 5 s; 70 °C for 10 s; 70 °C for 15 s; 70 °C for 20 s; 70 °C for 25 s; 72 °C for 5 s; 72 °C for 10 s; 72 °C for 15 s; 72 °C for 20 s; 72 °C for 25 s; 75 °C for 5 s; 75 °C for 10 s; 75 °C for 15 s; 75 °C for 20 s; 75 °C for 25 s | (1) |
| **Viral Pathogen** | **Viral Inactivation Observed** | **Temperature Monitoring** | **Temperature Reached (Methodology)** | **Author** |
| Cytomegalovirus (CMV) | Yes (except at 72°C for 1 s and 87°C for 1 s) | Yes | 72°C for 1 s; 72°C for 3 s; 72°C for 5 s; 72°C for 15 s; 87°C for 1 s, 87°C for 3 s; 87°C for 5 s | (11) |
| Cytomegalovirus (CMV) | Yes | Yes | 62 °C for 5 s; 62 °C for 15 s; 72 °C for 5 s; 72 °C for 15 s; 87 °C for 2 s; 87 °C for 5 s | (5) |
| Pseudorabies virus (PRV; HBV surrogate) | Yes | Yes | 72 °C for 16 s | (10) |
| Bovine viral diarrhea virus (BVDV; Hepatitis C Virus surrogate) | Yes | Yes | 72 °C for 16 s | (10) |
| Hepatitis A virus (HAV) | No, reduced but not eliminated | Yes | 72 °C for 16 s | (10) |
| Human immunodeficiency virus (HIV; marker virus) | Yes | Yes | 72 °C for 16 s | (10) |
| Porcine parvovirus (PPV; parvovirus B19 surrogate) | No | Yes | 72 °C for 16 s | (10) |

**References**

1. Escuder-Vieco D, Espinosa-Martos I, Rodríguez JM, Corzo N, Salcedo P, Montes MT, et al. High-temperature short-time pasteurization system for donor milk in a human milk bank setting. Front Microbiol. (2018) 9:926. doi: 10.3389/fmicb.2018.00926
2. Klotz D, Joellenbeck M, Winkler K, Kunze M, Huzly D, Hentschel R. High-temperature short-time pasteurisation of human breast milk is efficient in retaining protein and reducing the bacterial count. Acta Paediatr. (2017) 106:763–7. doi: 10.1111/apa.13768
3. Giribaldi M, Coscia A, Peila C, Antoniazzi S, Lamberti C, Ortoffi M, et al. Pasteurization of human milk by a benchtop high-temperature short-time device. Innov Food Sci Emerg Technol. (2016) 36:228–33. doi: 10.1016/j.ifset.2016.07.004
4. Manzardo OA, Toll LJ, Müller K, Nickel E, Jonas D, Baumgartner J, et al. A novel heat treatment protocol for human milk. Front Pediatr. (2022) 10:990871. doi: 10.3389/fped.2022.990871
5. Klotz D, Scherf M, Fuchs V, Jopling J, Kunze M, Winkler A, et al. High-temperature short-time treatment of human milk for bacterial count reduction. Front Pediatr. (2018) 6:359. doi: 10.3389/fped.2018.00359
6. Aceti A, Cavallarin L, Martini S, Giribaldi M, Vitali F, Ambretti S, et al. Effect of alternative pasteurization techniques on human milk’s bioactive proteins. J Pediatr Gastroenterol Nutr. (2020) 70:508–12. doi: 10.1097/MPG.0000000000002598
7. Dhar J, Fichtali J, Skura BJ, Nakai S, Davidson AGF. Pasteurization efficiency of a HTST system for human milk. J Food Sci. (1996) 61:569–73. doi: 10.1111/j.1365-2621.1996.tb13160.x
8. Morgan JN, Lin FJ, Eitenmiller RR, Barnhart HM, Toledo RT. Thermal destruction of Escherichia coli and Klebsiella pneumoniae in human milk. J Food Prot. (1988) 51:132–6. doi: 10.4315/0362-028X-51.2.132
9. Silvestre D, Miranda M, Muriach M, Almansa I, Jareño E, Romero FJ. Antioxidant capacity of human milk: effect of thermal conditions for the pasteurization. Acta Paediatr. (2008) 97:1070–4. doi: 10.1111/j.1651-2227.2008.00870.x
10. Terpstra FG, Rechtman DJ, Lee ML, Hoeij KV, Berg H, Van Engelenberg FAC, et al. Antimicrobial and antiviral effect of high-temperature short-time (HTST) pasteurization applied to human milk. Breastfeed Med. (2007) 2:27–33. doi: 10.1089/bfm.2006.0015
11. Goldblum RM, Dill CW, Albrecht TB, Alford ES, Garza C, Goldman AS. Rapid high-temperature treatment of human milk. J Pediatr. (1984) 104:380–5. doi: 10.1016/S0022-3476(84)81099-9
