# Supplementary material for: A scoping review of microbiological safety, bioactive components, nutrient retention, and feasibility of flash-heating expressed breastmilk: exploring potential use during infectious disease outbreaks
Source: Front Nutr. 2026 Oct 15;13:1926806. doi: 10.3389/fnut.2026.1926806 (PMC13717084; doi:10.3389/fnut.2026.1926806)
Supplement: Supplementary file 3 [file Table_3.docx]

**Supplementary Material**

**Supplementary Table 3. High Temperature Short Time (HTST) Effects on Retention (%) of Immunological and Nutrient Components**

| **Immunological Component(s) Assessed** | **Temperature (Reported in Methodology)** | **Notes on Degree of Retention^** | **Reference** |
| --- | --- | --- | --- |
| Lactoferrin | 72°C, 15 s | 23% | (1) |
| Lactoferrin | 72°C, 15 s | Preserved, no quantitative data reported | (2) |
| Lactoferrin | 72°C, 1 s | ~100%* | (3) |
| Lactoferrin | 72°C, 3 s | 87% |  |
| Lactoferrin | 72°C, 15 s | ~100%* |  |
| Lactoferrin | 87°C, 1 s | 75% |  |
| Lactoferrin | 87°C, 3 s | 75% |  |
| Lactoferrin | 87°C, 15 s | 70% |  |
| Lactoferrin | 62 °C, 5 s | 32% | (4) |
| Lactoferrin | 72°C, 15 s | 48% | (5) |
| Lactoferrin | 62°C, 5 s | 53% | (6) |
| Lactoferrin | 72°C, 5 s | 27% |  |
| Lactoferrin | 81°C, 5 s | 22% |  |
| Lysozyme | 72°C, 15 s | 97% | (7) |
| Lysozyme | 72°C, 1 s | ~100%* | (3) |
| Lysozyme | 72°C, 3 s | ~100%* |  |
| Lysozyme | 72°C, 15 s | ~100%* |  |
| Lysozyme | 87°C, 1 s | ~100%* |  |
| Lysozyme | 87°C, 3 s | ~100%* |  |
| Lysozyme | 87°C, 15 s | ~100%* |  |
| Lysozyme | 62 °C, 5 s | 72% | (4) |
| Lysozyme | 72°C, 15 s | 82% | (5) |
| Immunoglobulin A (IgA) | 72°C, 15 s | Preserved, no quantitative data reported | (2) |
| Immunoglobulin A (IgA) | 71°C, 72.8°C, and 74°C with duration varies 5.75-18.5 s (flow rates: 5.9, 12.3, and 18,9 mL/min) | 47–83% | (8) |
| Immunoglobulin A (IgA) | 70°C, 72°C, and 75°C, with duration varies at 5 s, 10 s, 15 s, 20 s, 25 s | 54-88% | (9) |
| Immunoglobulin A (IgA) | 72°C, 1 s | 100% | (3) |
| Immunoglobulin A (IgA) | 72°C, 3 s | 68% |  |
| Immunoglobulin A (IgA) | 72°C, 15 s | 84% |  |
| Immunoglobulin A (IgA) | 87°C, 1 s | 16% |  |
| Immunoglobulin A (IgA) | 87°C, 3 s | 11% |  |
| Immunoglobulin A (IgA) | 87°C, 15 s | 14% |  |
| Immunoglobulin A (IgA) | 72°C, 15 s | 74% | (5) |
| Immunoglobulin A (IgA) | 62 °C, 5 s | 95% | (4) |
| Secretory Immunoglobulin A (IgA) | 72°C, 15 s | ~78% | (1) |
| Secretory Immunoglobulin A (IgA) | 72°C, 15 s | 79.00% | (7) |
| Secretory Immunoglobulin A (IgA) | 62°C, 5 s | 82% | (6) |
| Secretory Immunoglobulin A (IgA) | 72°C, 5 s | 18.50% |  |
| Secretory Immunoglobulin A (IgA) | 81°C, 5 s | 0% |  |
| Immunoglobulin M (IgM) | 71°C, 72.8°C, and 74°C with duration varies 5.75-18.5 s (flow rates: 5.9, 12.3, and 18,9 mL/min) | 33–72.1% | (8) |
| Immunoglobulin M (IgM) | 70°C, 72°C, and 75°C, with duration varies at 5 s, 10 s, 15 s, 20 s, 25 s | 25-73% | (9) |
| Immunoglobulin G (IgG) | 71°C, 72.8°C, and 74°C with duration varies 5.75-18.5 s (flow rates: 5.9, 12.3, and 18,9 mL/min) | 35–79.1% | (8) |
| Immunoglobulin G (IgG) | 70°C, 72°C, and 75°C, with duration varies at 5 s, 10 s, 15 s, 20 s, 25 s | 87-100% | (9) |
| Nutrients Component(s) Assessed | Temperature (Reported in Methodology) | Notes on Degree of Retention^ | Reference |
| Bile Salt-Stimulated Lipase (BSSL) | 72°C, 15 s | Preserved, no quantitative data reported | (2) |
| Bile Salt-Stimulated Lipase (BSSL) | 70°C, 72°C, and 75°C, with duration varies at 5 s, 15 s, 25 s | 20% | (10) |
| Bile Salt-Stimulated Lipase (BSSL) | 72°C, 15 s | 0.18% | (7) |
| Bile Salt-Stimulated Lipase (BSSL) | 62 °C, 5 s | 0.80% | (4) |
| Bile Salt-Stimulated Lipase (BSSL) activity | 72°C, 15 s | 19% | (5) |
| Fat | 70°C, 72°C, and 75°C, with duration varies at 5 s, 10 s, 15 s, 20 s, 25 s | 96-100% | (10) |
| Lactose | 70°C, 72°C, and 75°C, with duration varies at 5 s, 10 s, 15 s, 20 s, 25 s | ~100% | (10) |
| Total Antioxidant Capacity | 75°C, 15 s | 100% | (11) |
| Vitamins | 70°C, 72°C, and 75°C, with duration varies at 15 s, 25 s | Vitamin A: ~100%  Vitamin B2: ~100%  Vitamin B3: ~92%  Vitamin B6: ~91%  Vitamin B12: ~100%  Vitamin E (α): ~92%  Vitamin E (γ): ~100%  Vitamin D: ~100% | (10) |
| Vitamins | 72°C, 1 s | Vitamin B1: 94%  Vitamin B2: ~100%*  Vitamin B6: ~100%*  Vitamin B9: 84%  Vitamin C: ~100%* | (3) |
| Vitamins | 72°C, 3 s | Vitamin B1: 88%  Vitamin B2: 97%  Vitamin B6: ~100%*  Vitamin B9: 61%  Vitamin C: ~100%* |  |
| Vitamins | 72°C, 15 s | Vitamin B1: 85%  Vitamin B2: 77%  Vitamin B6: 93%  Vitamin B9: 95%  Vitamin C: 95% |  |
| Vitamins | 87°C, 1 s | Vitamin B1: 81%  Vitamin B2: 91%  Vitamin B6: ~100%*  Vitamin B9: 83%  Vitamin C: ~100%* |  |
| Vitamins | 87°C, 3 s | Vitamin B1: 91%  Vitamin B2: 99%  Vitamin B6: ~100%*  Vitamin B9: 76%  Vitamin C: ~100%* |  |
| ^Degree of preservation was extracted as reported in the original studies. When not explicitly reported, it was calculated as the post-heating value divided by the pre-heating value and expressed as a percentage. Values presented only graphically were estimated from the published figures.  *Values initially exceeded 100% and are reported as~100%, reflecting post-heating increases, which may be attributable to the release of nutrients from binding proteins during heating. | | | |

**References**

1. Aceti A, Cavallarin L, Martini S, Giribaldi M, Vitali F, Ambretti S, et al. Effect of alternative pasteurization techniques on human milk’s bioactive proteins. J Pediatr Gastroenterol Nutr. (2020) 70:508–12. doi: 10.1097/MPG.0000000000002598
2. Baro C, Giribaldi M, Arslanoglu S, Giuffrida MG, Dellavalle G, Conti A, et al. Effect of two pasteurization methods on the protein content of human milk. Front Biosci (Elite Ed). (2011) 3:818–29. doi: 10.2741/e289
3. Goldblum RM, Dill CW, Albrecht TB, Alford ES, Garza C, Goldman AS. Rapid high-temperature treatment of human milk. J Pediatr. (1984) 104:380–5. doi: 10.1016/S0022-3476(84)81099-9
4. Klotz D, Joellenbeck M, Winkler K, Kunze M, Huzly D, Hentschel R. High-temperature short-time pasteurisation of human breast milk is efficient in retaining protein and reducing the bacterial count. Acta Paediatr. (2017) 106:763–7. doi: 10.1111/apa.13768
5. Kontopodi E, Boeren S, Stahl B, van Goudoever JB, van Elburg RM, Hettinga K. High-temperature short-time preserves human milk’s bioactive proteins and their function better than pasteurization techniques with long processing times. Front Pediatr. (2021) 9:798609. doi: 10.3389/fped.2021.798609
6. Manzardo OA, Toll LJ, Müller K, Nickel E, Jonas D, Baumgartner J, et al. A novel heat treatment protocol for human milk. Front Pediatr. (2022) 10:990871. doi: 10.3389/fped.2022.990871
7. Giribaldi M, Coscia A, Peila C, Antoniazzi S, Lamberti C, Ortoffi M, et al. Pasteurization of human milk by a benchtop high-temperature short-time device. Innov Food Sci Emerg Technol. (2016) 36:228–33. doi: 10.1016/j.ifset.2016.07.004
8. Dhar J, Fichtali J, Skura BJ, Nakai S, Davidson AGF. Pasteurization efficiency of a HTST system for human milk. J Food Sci. (1996) 61:569–73. doi: 10.1111/j.1365-2621.1996.tb13160.x
9. Escuder-Vieco D, Espinosa-Martos I, Rodríguez JM, Fernández L, Pallás-Alonso CR. Effect of HTST and Holder pasteurization on the concentration of immunoglobulins, growth factors, and hormones in donor human milk. Front Immunol. (2018) 9:2222. doi: 10.3389/fimmu.2018.02222
10. Escuder-Vieco D, Rodríguez JM, Espinosa-Martos I, Corzo N, Montilla A, García-Serrano A, et al. High-temperature short-time and Holder pasteurization of donor milk: impact on milk composition. Life. (2021) 11:114. doi: 10.3390/life11020114
11. Silvestre D, Miranda M, Muriach M, Almansa I, Jareño E, Romero FJ. Antioxidant capacity of human milk: effect of thermal conditions for the pasteurization. Acta Paediatr. (2008) 97:1070–4. doi: 10.1111/j.1651-2227.2008.00870.x
